# *O*^6^-methylguanine-DNA methyltransferase depletion and DNA damage in patients with melanoma treated with temozolomide alone or with lomeguatrib

**DOI:** 10.1038/sj.bjc.6605015

**Published:** 2009-04-14

**Authors:** A J Watson, M R Middleton, G McGown, M Thorncroft, M Ranson, P Hersey, G McArthur, I D Davis, D Thomson, J Beith, A Haydon, R Kefford, P Lorigan, P Mortimer, A Sabharwal, O Hayward, G P Margison

**Affiliations:** 1 Cancer Research UK Carcinogenesis Group, Paterson Institute for Cancer Research, Wilmslow Road, Manchester M20 9BX, UK; 2 Department of Medical Oncology, Churchill Hospital, University of Oxford, Oxford OX3 7LJ, UK; 3 Department of Medical Oncology, Christie Hospital, Wilmslow Road, Manchester M20 4BX, UK; 4 Newcastle Melanoma Unit, David Maddison Building, Newcastle, New South Wales 2300, Australia; 5 Peter MacCallum Cancer Institute, St Andrews Place, East Melbourne, Victoria 3002, Australia; 6 Austin Health, 145 Studley Road, Heidelberg, Victoria 3084, Australia; 7 Princess Alexandra Hospital, Ipswich Road, Woolloongabba, Queensland 4102, Australia; 8 Sydney Melanoma Unit, Royal Prince Alfred Hospital, Missenden Road, Camperdown, New South Wales 2050, Australia; 9 The Alfred Hospital, PO Box 315, Prahran, Victoria 3181, Australia; 10 Department of Medicine, Westmead Hospital, Westmead, New South Wales 2145, Australia; 11 Kudos Pharmaceuticals, 410 Cambridge Science Park, Milton Road, Cambridge, CB4 0PE, UK

**Keywords:** *O*^6^-methylguanine-DNA methyltransferase, lomeguatrib, temozolomide, melanoma

## Abstract

We evaluated the pharmacodynamic effects of the *O*^6^-methylguanine-DNA methyltransferase (MGMT) inactivator lomeguatrib (LM) on patients with melanoma in two clinical trials. Patients received temozolomide (TMZ) for 5 days either alone or with LM for 5, 10 or 14 days. Peripheral blood mononuclear cells (PBMCs) were isolated before treatment and during cycle 1. Where available, tumour biopsies were obtained after the last drug dose in cycle 1. Samples were assayed for MGMT activity, total MGMT protein, and *O*^6^-methylguanine (*O*^6^-meG) and N7-methylguanine levels in DNA. MGMT was completely inactivated in PBMC from patients receiving LM, but detectable in those on TMZ alone. Tumours biopsied on the last day of treatment showed complete inactivation of MGMT but there was recovery of activity in tumours sampled later. Significantly more *O*^6^-meG was present in the PBMC DNA of LM/TMZ patients than those on TMZ alone. LM/TMZ leads to greater MGMT inactivation, and higher levels of *O*^6^-meG than TMZ alone. Early recovery of MGMT activity in tumours suggested that more protracted dosing with LM is required. Extended dosing of LM completely inactivated PBMC MGMT, and resulted in persistent levels of *O*^6^-meG in PBMC DNA during treatment.

Temozolomide (TMZ) is an oral methylating agent used in the treatment of primary CNS tumours and melanoma. The drug hydrolyses spontaneously to methyltriazenoimidazolecarboxamide. This rapidly decomposes to a reactive methylating species that attacks DNA to generate a number of adducts, the most common being N7-methylguanine (N7-meG). Although constituting only 6% of the total products, *O*^6^-methylguanine (*O*^6^-meG) is responsible for the majority of the toxic effects of TMZ (Middleton and Margison, 2003). *O*^6^-meG is a promutagenic, recombinogenic and cytotoxic DNA lesion (Gerchman and Ludlum, 1973; Brennand and Margison, 1986). These biological effects require two rounds of DNA replication (Kaina *et al*, 1997) and a functional mismatch repair (MMR) system is obligatory for cell killing (Karran and Bignami, 1994).

The DNA repair protein *O*^6^-methylguanine-DNA methyltransferase (MGMT) can remove the methyl group from *O*^6^-meG in DNA and hence can protect cells against the toxic effects of this TMZ-induced damage (Middleton and Margison, 2003).

Many tumours express high levels of MGMT (Chen *et al*, 1992; Citron *et al*, 1994) and dose-limiting toxicities occur in the bone marrow, which expresses low levels of MGMT (Gerson *et al*, 1985). The inactivation of MGMT might thus be expected to increase the toxic effects of chemotherapy in tumours to a greater extent than in the bone marrow, and hence improve the therapeutic index.

Combinations of methylating agents with nitrosoureas have been used to attenuate MGMT activity. The methylating agent generates *O*^6^-meG in DNA and this consumes MGMT in being repaired, rendering the cell susceptible to the nitrosourea (Lee *et al*, 1993; Smith *et al*, 1996; Schold *et al*, 2000). Such combinations proved to be unacceptably toxic in clinical trials (Gerard *et al*, 1993; Clemons *et al*, 2003). The development of non-toxic MGMT pseudosubstrates has enabled this strategy to be revisited. *O*^6^-meG itself was shown to be very inefficient (Gerson *et al*, 1992) but its analogues, *O*^6^-benzylguanine (Dolan *et al*, 1992) and lomeguatrib (LM; 6-[4-bromo-2-thienyl]methoxypurin-2-amine) (Middleton *et al*, 2000a; Ranson *et al*, 2006), are considerably more potent MGMT inactivators and currently in clinical trials.

We undertook a phase II trial of LM and TMZ (LM/TMZ) to establish whether inactivation of MGMT by the former might increase the efficacy of TMZ in patients with advanced melanoma. The clinical outcome of the LM/TMZ combination was disappointing, with efficacy similar to that of standard dose TMZ (Ranson *et al*, 2007). Here we present the detailed pharmacodynamic (PD) analysis from that study, which used a 5-day LM/TMZ treatment schedule, and show that there was post-LM/TMZ recovery of tumour MGMT activity. We hypothesised that this would have resulted in the repair of *O*^6^-meG before the two rounds of DNA replication required for cytotoxicity occurred. We have therefore also assessed if extended dosing (10–14 days) with LM could maintain MGMT inactivation. Although the intention was to assess this in tumour material, only peripheral blood mononuclear cells (PBMCs) were available from this study. We undertook to quantify *O*^6^-meG in PBMC DNA as a measure of the effect of MGMT inactivation. Since the levels of the base would be affected by cell turnover, we quantified the major DNA lesion 7-meG as a referent, bearing in mind that this may be affected by the expression levels of the DNA repair enzyme, 3-alkyladenine-DNA glycosylase (AAG). The accompanying paper presents the clinical outcome for the extended LM study.

## Patients and methods

### Patients

Patients with metastatic melanoma were eligible for the studies, provided that they had not previously received systemic chemotherapy. The first trial was a multi-centre randomised study in which 104 patients were allocated one of the two treatments: LM/TMZ or TMZ alone. Patients experiencing disease progression on TMZ alone were permitted to change to the LM/TMZ combination, allowing assessment of whether LM could reverse resistance to TMZ. The protracted LM study was based on the PD observations described below and involved 34 patients, all treated with LM/TMZ, of whom 10 contributed PD samples.

The studies were conducted in accordance with the International Conference on Harmonisation of Good Clinical Practice guidelines and the Declaration of Helsinki Principles. The trial was approved by independent ethics committees according to national and local requirements at each trial centre. All patients gave informed, written consent.

### Treatment

LM enteric-coated 10 mg capsules were obtained from Kudos Pharmaceuticals (Cambridge, UK), and TMZ was purchased from Schering Plough Ltd (Welwyn Garden City, UK) as 5, 20, 100 and 250 mg capsules.

Patients assigned LM/TMZ received LM 40 mg per day p.o. for 5 consecutive days every 4 weeks for up to six cycles. Those among the last 20 patients who were randomised to LM/TMZ received LM 60 or 80 mg per day. Temozolomide was administered at 125 mg m^−2^ per day p.o. 2 h after LM. On the TMZ-alone arm, patients received a starting dose of 200 mg m^−2^ per day, administered on days 1–5 of a 4-week cycle. Patients receiving LM/TMZ after progressing on TMZ alone were treated as above, although the TMZ dose was adjusted to take into account any earlier dose reductions on TMZ.

In the protracted LM study, patients received LM 40 mg per day p.o. twice daily for 10 or 14 consecutive days every 4 weeks for up to six cycles. TMZ was administered at 75–125 mg m^−2^ per day p.o. 2 h after the first LM dose on days 1–5 of each cycle.

### PBMC and tumour sampling

Peripheral blood mononuclear cell samples were obtained before treatment, 6 and 24 h after first LM dosing on day 1 and between 0 and 72 h after last LM dose on day 5 of cycle 1. In the protracted LM study, PBMC samples were obtained before and 4 h after LM dosing on day 1 and following last dose on day 10 or 14. Venous blood (5–10 ml) was collected into tubes containing 100 μl 0.5 M EDTA and stored on ice for up to 4 h before isolation of PBMC. Additional samples were drawn in patients in whom pharmacokinetics (PK) were evaluated, these being 2, 4 and 8 h after LM administration on day 1 of cycle 1. Tumour biopsies were obtained by excision biopsy between 0 and 72 h after completion of the first cycle of treatment. The biopsies were snap-frozen on dry ice and stored at −80°C.

**Figure 1:**
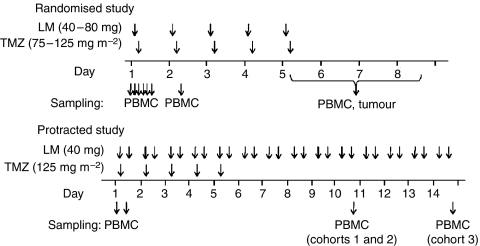
Schedules of treatment (above time lines) and sampling (below time lines) for the randomised and protracted studies as indicated. Further details are provided in the Patients and Methods section.

A schematic diagram of the above treatment and sampling schedules is shown in Figure 1.

### MGMT activity

*O*^6^-methylguanine-DNA methyltransferase activity in PBMC and tumour cell sonicates was determined as previously described (Watson and Margison, 2000).

### Total MGMT protein

Total, that is, active and inactivated, MGMT protein was determined in cleared tumour sonicates by ELISA. Briefly, 96-well plates were coated with mouse anti-MGMT monoclonal antibody (Abcam, Cambridge, UK) overnight at 4°C. After washing with PBS, blocking buffer (10% horse serum, 0.1% Tween 20 in PBS) was added to each well and incubated for 2 h at room temperature. Blocking buffer was removed and the following 1 h room temperature incubations were performed in sequence with washing between additions: tumour sonicate, rabbit anti-MGMT polyclonal antibody (Lee *et al*, 1996), goat anti-rabbit HRP antibody (Ely, UK). Finally, plates were washed with PBS, Western Lightning reagent (PerkinElmer, Beaconsfield, UK) was added and luminescence was detected using a Tecan GENios plate reader. *O*^6^-ethylguanine-DNA methyltransferase values were extrapolated from a purified human MGMT standard curve using Magellan v3 software.

### N7-methylguanine

N7-methylguanine in DNA was quantified by an immunoslot blot method and using the antibodies as previously described (Harrison *et al*, 2001) with one modification: instead of whole tissue/cells, DNA was isolated from sonicates.

### *O*^6^-methylguanine

*O*^6^-methylguanine in DNA was quantified using a modification of the standard MGMT activity assay procedure (Watson and Margison, 2000). Incrementally increasing amounts of the DNA samples were pre-incubated with a standard amount of purified recombinant human MGMT (Elder *et al*, 1994) and residual activity was then determined. *O*^6^-meG in DNA stoichiometrically inactivates MGMT. Thus the amount of *O*^6^-meG in the DNA sample equals the amount of inactivation of the purified MGMT. For the protracted LM study a more sensitive assay was used. Briefly, the residual MGMT activity was determined by incubation with a [^32^P]-labelled oligonucleotide containing *O*^6^-meG within a *Pst*I (New England Biolabs, Hitchin, Hertfordshire, UK) recognition site attached by 3′biotin to the wells of streptavidin-coated plates. Repair of the *O*^6^-meG by the residual active MGMT results in de-protection of the restriction site and, upon incubation with *Pst*I, release of a short radiolabelled oligonucleotide fragment into the supernatant. Thus residual MGMT activity is proportional to the amount of radioactivity present in the supernatant, quantified in a TopCount machine (PerkinElmer).

## Results

The randomised study recruited 104 patients split equally into two treatment arms. Of the 52 patients initially treated with TMZ alone, 27 were transferred to LM/TMZ at progression. Of the first 84 patients, those assigned to the LM/TMZ arm received 40 mg per day LM. The PD results obtained in the course of the study indicated not quite complete MGMT inactivation and this forced us to increase the daily LM dose.

Some samples were missed at the study site, deteriorated in transit or were insufficient to perform all of the assays intended. For the principal time points on the first day of treatment, data were obtained from over 60% of the patients. Tumour biopsies were taken in 25 instances: 14 from patients on LM/TMZ, 5 on TMZ alone and 6 from patients on LM/TMZ having progressed on TMZ alone.

Samples collected from 10 patients on protracted schedules of LM were available for analysis. One patient was withdrawn on day 8; since the post-treatment sample was missing the other samples collected from this patient were not analysed. In addition, not all samples were sufficient to quantitate MGMT activity and DNA methylation.

### MGMT activity in PBMC

**Figure 2:**
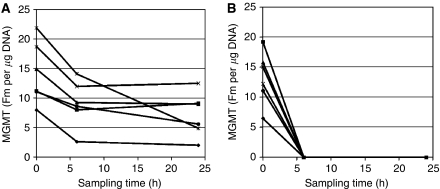
MGMT activity in PBMC sampled at the times indicated from patients treated with (**A**) TMZ alone and (**B**) LM/TMZ. Further details are provided in the Patients and Methods section.

In PBMC, MGMT activity before treatment ranged between 2.3 and 51.6 fmol μg^−1^ DNA. The mean was similar for all the treatment groups but was lower before starting LM/TMZ at progression (Table 1). Levels fell more rapidly and completely with combination therapy than with TMZ alone. At 6 and 24 h after LM dosing, PBMC taken from patients on LM/TMZ had no detectable MGMT activity. All TMZ-treated patients had residual activity, with a mean of 88 and 74% of pre-treatment levels at 6 and 24 h respectively (Figure 2). Previous studies of MGMT in PBMC following TMZ alone showed progressive, but incomplete inactivation (maximally ∼30% of activity remaining) over the 5-day schedule (Lee *et al*, 1994), broadly agreeing with these observations. MGMT activity was undetectable in all samples taken at the end of prolonged treatment (ie on day 10 or 14; Table 1).

### MGMT activity in tumour

**Figure 3:**
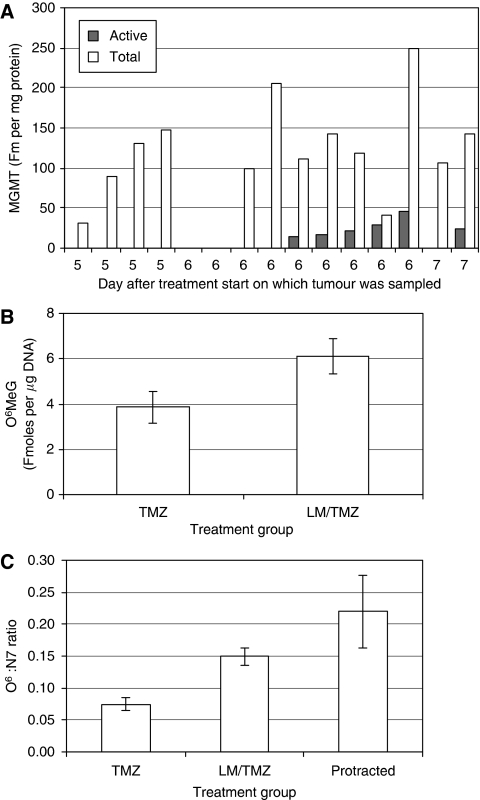
(**A**) Active (filled columns) *vs* total (open columns) MGMT protein in extracts of individual tumour biopsies taken from LM/TMZ patients on days 5 (four samples), 6 (nine samples) and 7 (two samples) of treatment cycle 1. Note that all samples were analysed for both parameters and several of the samples had levels that were lower than the detection limit. (**B**) *O*^6^-MeG and (**C**) its ratio to N7-meG levels in DNA extracted from PBMC taken at the end of treatment in cycle 1. Error bars represent the s.e.m. for 9. TMZ, 13 LM/TMZ and 6 protracted LM/TMZ DNA samples. Figure 1contains a more comprehensive description of the treatment and sampling schemes.

Among the first 84 patients in the randomised trial, tumour biopsies were obtained on days 6–8 of cycle one from 3 patients on TMZ alone and from 9 on LM/TMZ (Table 2). Unexpectedly, residual MGMT activity was detected in five of these nine samples. The dose of LM given to subsequent patients was increased from 40 to 60 mg and then 80 mg to try to achieve complete tumour MGMT inactivation. Despite this, residual activity was detectable in six of seven tumour biopsies taken from LM/TMZ patients on day 6 or 7. *O*^6^-methylguanine-DNA methyltransferase was completely inactivated in all four samples taken on day 5, within hours of completing treatment (Table 2; Figure 3A). One of the two samples obtained on day 5 of TMZ alone had residual MGMT activity (Table 2).

### Total MGMT protein

Total MGMT protein was determined in 15 tumour biopsies taken following LM/TMZ (Figure 3A). *O*^6^-methylguanine-DNA methyltransferase protein was detectable in all but two samples (both taken on day 6). There was complete inactivation of MGMT in all biopsies taken at cessation of treatment, on day 5. In the biopsies taken on day 6 or 7 the amount of protein that was active ranged from 0 to 73%. There was no correlation between the day of biopsy and total MGMT protein levels.

### DNA methylation damage

Mean pre-dose levels of N7-meG were below the lower limit of quantitation (0.3 fmol μg^−1^ DNA) in 43 of the 49 PBMC DNA samples isolated from previously untreated patients (Table 3). N7-methylguanine was detectable in all five samples analysed before LM/TMZ treatment from patients progressing on TMZ. N7-methylguanine levels in PBMCs rose after treatment, with the highest levels measured on day 6, the day after TMZ dosing was completed. Patients treated with TMZ alone had the highest levels, consistent with their daily dose of 200 mg m^−2^ as compared to the 75–125 mg m^−2^ administered within LM/TMZ. In patients treated with protracted LM schedules, the mean N7-meG level for PBMC samples taken at day 10 (16.1±2.6 fmol μg^−1^ DNA, *n*=3) was higher than that for those taken at day 14 (6.9±4.8 fmol μg^−1^ DNA, *n*=4), consistent with the previous observation that levels reduce over time. This is most likely due to cell turnover as a consequence of toxicity and/or repair of N7-meG by AAG. Levels of N7-meG in post-treatment tumour samples were consistent with those observed in PBMC (Table 4).

Pre-dose levels of *O*^6^-meG were less than the lower limit of quantitation (0.5 fmol μg^−1^ DNA) in all PBMC DNA samples analysed (Table 5). Mean *O*^6^-meG levels in DNA from post-treatment PBMC samples were lower with TMZ alone than with LM/TM (Table 5; Figure 3B; *P*⩽0.05). The ratio of *O*^6^-meG to N7-meG in the same sample was twice as high on LM/TMZ as with TMZ alone (Figure 3C; *P*=0.0005). Where longer courses of LM were given, mean *O*^6^-meG levels in DNA from PBMC samples were higher at day 10 than at day 14 (Table 5). Since levels of N7-meG were also reduced and MGMT activity was not detectable, this is probably due to cell turnover, that is, dilution of *O*^6^-meG in DNA by routine replacement of PBMC in the blood.

## Discussion

We have previously reported that the clinical activity of LM/TMZ was similar to that of TMZ alone, and that no patients progressing on the latter subsequently responded to LM/TMZ (Ranson *et al*, 2006). Here we present the PD findings of this study, together with the subsequent protracted LM dose study. Levels of MGMT activity in PBMC were comparable with those previously reported (Ranson *et al*, 2006, 2007). There was slow and incomplete inactivation following treatment with TMZ and more rapid, total, depletion with LM/TMZ, consistent with our previous trial (Ranson *et al*, 2006).

N7-methylguanine was detectable, albeit at very low levels, in 12% of the pre-treatment PBMCs, consistent with previous measurements in the blood of non-smokers (Harrison *et al*, 2001). This may reflect exposure of these individuals to environmental or endogenous methylating agents and/or deficiency in AAG. Post-treatment levels were highest in the patients treated with TMZ alone, due to the higher dose of TMZ given. Importantly, N7-meG levels per unit dose were similar, consistent with the observation that LM had no effect on the PK of TMZ in those patients on LM/TMZ. We found increased levels of N7-meG in all of the TMZ progressors before they started on LM/TMZ: these patients had already undergone at least two treatment cycles with TMZ and the relatively slow repair of N7-meG is well documented (Lawley *et al*, 1986).

Despite the much lower dose of TMZ administered in combination with LM, the levels of *O*^6^-meG were significantly greater in the PBMC of patients on combination therapy. Thus the ratio of *O*^6^/N7-meG was significantly higher in these patients than those on TMZ alone, confirming that effective and prolonged inactivation of MGMT, as was the case in PBMC, results in increased levels of the potentially toxic lesion *O*^6^-meG in DNA. These increased levels of *O*^6^-meG in PBMC are likely to reflect increasing levels in blood progenitor cells and hence the increased haematological toxicity in patients receiving LM/TMZ. Unfortunately, we were unable to compare levels of *O*^6^-meG in tumour DNA between the treatment groups due to the low amounts of tumour DNA available and the sensitivity of the assay.

In the phase 1 study of LM/TMZ, we observed complete inactivation of tumour MGMT after a single dose of LM in three of five patients with melanoma and >96% inactivation in the other two. In that trial, tumours were biopsied before and 4 h after the first LM dose. In the current study, to avoid the possibility of inherent differences between two tumour biopsies (as observed in earlier studies, Egyházi *et al*, 1997) we measured total (active and inactive) MGMT using an ELISA assay and active MGMT using our functional activity assay. None of the four tumours sampled on day 5 of LM/TMZ treatment had residual MGMT activity, although ELISA showed that MGMT protein was present in all cases. However, we found residual MGMT activity in the majority of tumour biopsies taken at later times after the end of the first LM/TMZ cycle, even when the daily dose of LM was increased.

It appears therefore that MGMT activity can recover rapidly after LM/TMZ treatment. This finding is significant, in that *O*^6^-meG is toxic only after two rounds of DNA replication, so that MGMT-mediated repair occurring before this (Kaina *et al*, 1997) or even after one round of DNA replication (Lips and Kaina 2001) will diminish cytotoxicity. Recovery of MGMT activity after dosing may not mean that complete repair of *O*^6^-meG has taken place, at least not across the whole tumour. We have found MGMT activity along with its substrate DNA lesion, *O*^6^-meG, in the same melanoma biopsy in a previous trial (Middleton *et al*, 2000b). This observation could reflect ongoing synthesis and inactivation in all cells, or adducts persisting in the fraction of cells in which MGMT recovery has not begun.

The critical factor for toxicity is whether recovery of MGMT activity takes place before DNA replication across a template containing *O*^6^-meG. One report, based on a study of 83 primary and metastatic malignant melanomas, indicates a potential doubling time (*T*_pot_) of human melanoma of 7.2 days (range 3.5–41 days) (Rew and Wilson, 2000). Human A375M melanoma cells and many of the other tumour cell lines studied in culture divide about every 24 h. The low cell-doubling rate in patients' tumours and the observed rapid recovery of MGMT activity suggest repair of *O*^6^-MeG may well precede two rounds of DNA replication, in contrast to the situation in cell culture and tumour xenograft models. It also contrasts with the reported doubling time of 24 h for the transit cell population of human bone marrow (Rew and Wilson, 2000; Begg, 2002), a factor that probably explains the increased haematological toxicity of combination therapy.

To test whether more protracted MGMT inactivation would enhance the effectiveness of TMZ, we conducted a study to examine the effect of an LM/TMZ combination with extended LM dosing to 10 or 14 days. The clinical outcome, reported in detail in the accompanying paper, showed no benefit, despite the continued suppression of MGMT activity and consequent persistence of *O*^6^-meG, at least in PBMC and PBMC DNA. The increased haematological toxicity is indicative of the importance of *O*^6^-meG, and it is reasonable to speculate that factors downstream of *O*^6^-meG, such as MMR and anti-apoptotic factors, and/or other TMZ-induced DNA lesions may have decisive functions in the resistance of melanoma to TMZ. In addition, with respect to DNA repair processes, recent studies show that a wide range of such processes are upregulated in poor prognosis melanoma (Kauffmann *et al*, 2008). More specifically, the effects of targeting the base excision repair pathway by blocking the processing of apurinic sites (Yan *et al*, 2007) or single-strand breaks through inhibition of poly-ADP-ribose polymerase activity (Naumann *et al*, 2009) are currently being assessed in clinical trials. Improving response rates in melanoma may rest on modulating these and other factors, in combination with the suppression of MGMT activity.

## Figures and Tables

**Table 1:** MGMT activity (fmol *μ*g^−1^ DNA) in extracts of PBMC samples from patients in the treatment groups indicated and as described in the Patients and Methods section

**Sampling time**	**LM/TMZ, 40 mg/day**	**LM/TMZ, 60–80 mg/day**	**TMZ alone**	**LM/TMZ on progression**	**Protracted LM/TMZ**
*Pre-treatment*					
*N*	28	7	30	9	7
Mean	16.5	12.6	16.9	9.9	13.9
Range	2.30–48.3	7.40–18.3	4.7–51.6	3.7–15.7	7–15.5
					
*2 h*					
*N*	3	—	—	4	—
Mean	6.43	—	—	1.10	—
Range	0–15.7	—	—	0–4.4	—
					
*4 h*					
*N*	2	—	—	3	6
Mean	0	—	—	0	0.6
Range		—	—	—	0–3.6
					
*6 h*					
*N*	27	6	27	7	—
Mean	0	0	14.4	0	—
Range	—	—	2.10–51.1	—	—
					
*8 h*					
*N*	2	—	—	3	—
Mean	0	—	—	0	—
Range	—	—	—	—	—
					
*24 h*					
*N*	26	3	26	4	—
Mean	0	0	12.5	0	—
Range	—	—	2.00–30.9	—	—
					
*End of treatment*					
*N*	—	—	—	—	4
Mean	—	—	—	—	0

Abbreviations: LM=lomeguatrib; TMZ=temozolomide.

**Table 2:** MGMT activity (fmol *μ*g^−1^ DNA) in extracts of tumour samples from patients in the treatment groups indicated and as described in the Patients and Methods section

**Sampling time**	**LM/TMZ, 40 mg/day^a^**	**LM/TMZ, 60–80 mg/day^a^**	**TMZ alone**
*Day 5*			
*N*	—	4	2
Mean	—	0	1.38
Range	—	—	0–2.8
			
*Day 6*			
*N*	6	6	2
Mean	0.64	2.79	1.23
Range	0–1.6	0–6.1	0–2.5
			
*Day 7*			
*N*	2	1	1
Mean	0.4	5.6	0
Range	0–0.8	—	—
			
*Day 8*			
*N*	1	—	—
Value	4.5	—	—

Abbreviations: LM=lomeguatrib; TMZ=temozolomide.

a Treated first line or after progression on TMZ alone.

**Table 3:** Levels of N7-meG (fmol *μ*g^−1^ DNA) in DNA extracted from PBMC samples from patients in the treatment groups indicated and as described in the Patients and Methods section

**Sampling time**	**LM/TMZ**	**TMZ alone**	**LM/TMZ at progression**	**Protracted LM/TMZ**
*Pre-dose*				
*N*	22	20	5	7
Mean	0.02	0.19	3.56	0.33
Range	0–0.5	0–3.0	1.5–6.1	0–0.5
				
*Day 5*				
*N*	1	5	1	—
Mean	38.0	52.9	51.0	—
Range	—	41.6–80.1	—	—
				
*Day 6*				
*N*	12	8	1	—
Mean	43.7	55.9	49.4	—
Range	29.7–64.2	34.1–79.3	—	—
				
*Day 7*				
*N*	10	5	3	—
Mean	35.9	43.6	35.3	—
Range	18.4–55.5	34.7–52.6	20.9–51.3	—
				
*Day 8*				
*N*	3	4	1	—
Mean	24.3	42.8	34.4	—
Range	17.7–29.2	28.3–64.7	—	—
				
*Day 10*				
*N*	—	—	—	3
Mean	—	—	—	16.1
Range	—	—	—	13.1–18.0
				
*Day 14*				
*N*	—	—	—	4
Mean	—	—	—	6.9
Range	—	—	—	3.6–13.9

Abbreviations: LM=lomeguatrib; TMZ=temozolomide.

**Table 4:** Levels of N7-meG (fmol *μ*g^−1^ DNA) in DNA extracted from tumour samples from patients in the treatment groups indicated and as described in the Patients and Methods section

**Sampling time**	**LM/TMZ**	**TMZ alone**	**LM/TMZ on progression**
*Day 6*			
*N*	4	1	2
Mean	36.0	40.1	50.6
Range	27.8–49.6	—	47.9–53.2
			
*Day 7*			
*N*	1	1	1
Value	38.8	40.4	33.3
			
*Day 8*			
*N*	1	—	—
Value	25.6	—	—

Abbreviations: LM=lomeguatrib; TMZ=temozolomide.

**Table 5:** Levels of *O*^6^-meG (fmol *μ*g^−1^ DNA) in DNA extracted from PBMC samples from patients in the treatment groups indicated and as described in the Patients and Methods section

**Sampling time**	**LM/TMZ**	**TMZ Alone**	**Protracted LM/TMZ**
*Pre-dose*			
*N*	—	—	4
Mean	—	—	0
			
*Day 5–8*			
*N*	13	9	—
Mean	6.1	3.9	—
Range	2.3–10.9	1.3–7.1	—
			
*Day 10*			
*N*	—	—	4
Mean	—	—	3.3
Range	—	—	2.2–5.4
			
*Day 14*			
*N*	—	—	4
Mean	—	—	1.4
Range	—	—	0.9–2.2

Abbreviations: LM=lomeguatrib; TMZ=temozolomide.
